# High-salt diet modulates endocrine regulation between cortisol and FGF23

**DOI:** 10.1007/s00424-026-03211-x

**Published:** 2026-09-19

**Authors:** Matthias B. Moor, Michael S. Sagmeister, Ana Crastin, Klaudia Kopper, Antoinette Pechère-Bertschi, Rowan S. Hardy, Eric Feraille, Daniel G. Fuster, Johannes Loffing, Ganesh Pathare

**Affiliations:** 1 Department of Nephrology and Hypertension, Inselspital, Bern University Hospital, University of Bern, Bern, Switzerland; 2 Swiss National Centre of Competence in Research “Kidney Control of Homeostasis”, Zurich, Switzerland; 3 College of Medicine and Health, Institute of Applied Health Research, University of Birmingham, Birmingham, B15 2TT UK; 4 MRC Arthritis Research UK Centre for Musculoskeletal Ageing Research, University of Birmingham, Birmingham, B15 2TT UK; 5 Department of Biomedical Sciences, School of Infection, Inflammation & Immunology, College of Medicine and Health, University of Birmingham, Birmingham, B15 2TT UK; 6 Institute of Anatomy, University of Zurich, Zurich, Switzerland; 7 Service of Nephrology and Hypertension, University Hospital Geneva, Geneva, Switzerland; 8 Department of Cell Physiology and Metabolism, University of Geneva, Geneva, Switzerland; 9 Institute of Veterinary Anatomy, University of Zurich, Zurich, Switzerland; 10 Bone-Kidney Axis and Regeneration Laboratory, Department of Infectious Diseases and Public Health, Jockey Club College of Veterinary Medicine and Life Sciences, City University of Hong Kong, Kowloon Tong, Hong Kong SAR China

**Keywords:** High-salt-diet, Cortisol (corticosterone), FGF23, Bone and kidney

## Abstract

Excessive dietary salt intake is a global health concern, affecting cardiovascular, renal, and bone health. While the renin–angiotensin–aldosterone system (RAAS) is a known regulator of dietary salt-induced hormonal responses, the impact of adrenal cortisol remains unclear. Here, we performed a retrospective analysis in individuals (*n* = 321) consuming a random diet. Dietary salt intake positively correlated with urinary cortisol and inversely correlated with plasma fibroblast growth factor 23 (FGF23), a bone-derived hormone regulating phosphate and vitamin D homeostasis. Controlled salt diets in healthy individuals confirmed a dose-dependent increase in urinary cortisol and suppression of plasma FGF23. In mice, oral corticosterone, a cortisol analogue, reduced circulating FGF23 levels. RNA-seq analysis of corticosterone-treated MC3T3 osteoblasts identified suppression of FGF23 via glucocorticoid receptor activation, anti-inflammatory pathways, and reduced osteoblast activity. Our findings provide evidence for an endocrine cascade in which high salt intake elevates cortisol signaling and suppresses FGF23.

**Supplementary Information:**

The online version contains supplementary material available at https://doi.org/10.1007/s00424-026-03211-x.

## Introduction

Excessive dietary salt intake, a hallmark of the Western diet, is a global health concern associated with cardiovascular disease, bone loss, and autoimmune disorders, contributing significantly to morbidity and mortality [1–3]. Beyond its well-established effects on blood pressure, high salt intake disrupts metabolic signaling and hormonal responses [3]. The renin–angiotensin–aldosterone system (RAAS) is the primary hormonal pathway influenced by dietary salt [4]. Notably, adrenal-derived cortisol is known for its role in stress response and immunosuppression, but it may also contribute to salt homeostasis [5, 6]. However, the mechanisms underlying this interaction and its impact on the pathophysiology of high salt intake are not yet fully understood.

Fibroblast growth factor 23 (FGF23), a bone-derived hormone, is central to phosphate and vitamin D homeostasis [7]. Elevated FGF23 is implicated in cardiovascular and kidney diseases, while its deficiency causes aging-like phenotypes in animal models [8–10]. FGF23 exerts its biological effects by binding to FGF receptors (FGFRs) in the presence of the co-receptor Klotho, a transmembrane protein predominantly expressed in the kidney [11]. Klotho abundance is regulated by physiological stimuli including osmotic stress and dehydration [12]. Distal tubular Klotho governs calcium reabsorption, while proximal tubular Klotho regulates phosphate homeostasis and prevents aging-like phenotypes in mice [11, 13]. Sodium transport regulatory proteins along the nephron are closely linked to FGF23-Klotho axis, and disruption of tubular sodium handling consistently alters circulating FGF23 levels [14–18].

Recent studies suggest a link between salt intake and circulatory FGF23 levels in humans and mice, but the mechanisms remain incompletely understood [14, 19, 20]^.^.Although extracellular sodium can directly regulate FGF23 production in osteoblasts [21], dietary high-salt intake under physiological conditions does not substantially alter plasma sodium concentrations [21]. Instead, it primarily induces changes in hormonal signaling and renal sodium-handling pathways, including the RAAS [4]. Dietary high-salt intake has been shown to suppress circulating FGF23 levels, an effect that has been proposed to involve aldosterone-mediated regulation of FGF23 [19, 20]. However, dietary salt intake induces broader hormonal adaptations beyond changes in the RAAS, including alterations in cortisol signaling [5]. Given the emerging role of cortisol in salt homeostasis and its established effects on bone remodeling through glucocorticoid receptors (GR), the potential interplay between dietary salt intake, cortisol, and FGF23 warrants further investigation.

Glucocorticoids have also been implicated in the regulation of FGF23, although the reported effects have been inconsistent. Earlier studies reported that exposure to synthetic glucocorticoids, including dexamethasone and prednisolone, increased FGF23 expression in cell and animal models [22]. In contrast, subsequent studies using similar synthetic glucocorticoids reported suppression of FGF23 expression in cell and animal models [23]. These conflicting findings suggest that the effects of glucocorticoids on FGF23 may depend on the specific glucocorticoid, experimental context, or duration of exposure. However, the role of endogenous glucocorticoid hormones, particularly corticosterone in rodents and cortisol in humans, in the regulation of FGF23 remains incompletely understood.

Here, we hypothesize that dietary salt intake may modulate endocrine regulation between cortisol and FGF23. To test this, we analyzed human cohort data and data from healthy individuals with and without dietary salt intervention and performed mechanistic experiments in mice and osteoblast cell lines. Our findings provide evidence for an endocrine cascade whereby dietary salt intake is associated with increased cortisol signaling and suppression of FGF23.

## Materials and methods

### Human cohort analysis

We retrospectively analyzed data from the Bern Kidney Stone Registry (BKSR), a previously described single-center observational cohort of adult kidney stone formers. Detailed information regarding recruitment procedures, inclusion and exclusion criteria, clinical characteristics, and phenotyping has been published previously [24]. For the present study, 321 participants with available measurements of urinary sodium, urinary steroids, and plasma cFGF23 were included. Plasma C-terminal FGF23 (cFGF23) levels were quantified using ELISA. Steroids in urinary samples were quantified using mass spectrometric analysis on a gas chromatograph 7890A coupled to a mass-selective Hewlett-Packard 5975C detector. Urinary cortisol measurements were available for 319 participants. Associations between urinary sodium, cortisol/cortisone, and plasma FGF23 levels were assessed using generalized linear models, after square-root or log transformation as appropriate based on residuals evaluation. Adjustments were made for age, sex, estimated glomerular filtration rate (creatinine CKD-EPI) and body mass index.

### Dietary salt intervention study

Urine and serum samples were obtained from a previously published prospective, crossover-controlled dietary intervention study in 16 healthy male volunteers [25]. For the present secondary analysis, archived serum and urine samples from 12 of the 16 original participants were available for the measurements performed in this study. Participants were normotensive men aged 18–30 years recruited among medical students of the Faculty of Medicine of Geneva, with plasma K⁺ ≤ 4.8 mmol/L, plasma creatinine ≤ 100 μmol/L, and no history of cardiac, renal, or endocrine disease. Exclusion criteria included hypertension (mean office blood pressure ≥ 140/90 mmHg after rest), use of anti-inflammatory drugs, diuretics, or corticosteroids, and a history of severe allergy. The study was conducted between 2016 and 2018 at the University Hospitals of Geneva, Switzerland, and was approved by the University Hospital Ethical Committee (2016–01779); written informed consent was obtained from all participants in accordance with the Declaration of Helsinki. Detailed information regarding dietary standardization procedures and compliance monitoring has been reported previously.

Detailed information regarding participant characteristics, inclusion and exclusion criteria, dietary standardization procedures, and compliance monitoring has been reported previously [25]. Participants underwent three 7-day dietary interventions: low-salt diet (LSD, 3 g NaCl/day), normal-salt diet (NSD, 6 g NaCl/day), and high-salt diet (HSD, 15 g NaCl/day), with constant potassium intake. Serum and 24-h urine samples were collected after each dietary phase. Concentrations of urinary free cortisol were measured using an ELISA (Diametra), whereas iFGF23 and cFGF23 levels were measured using an ELISA (Quidel). Changes in plasma hormone levels and urinary parameters across the dietary interventions were compared using repeated-measures one-way ANOVA (*n* = 12).

### Mouse experiments

C57BL/6 male mice aged 10–12 weekswere housed in standard conditions with ad lib access to standard chow and water. All animal experiments were performed in accordance with the guidelines and regulations approved by the University of Birmingham. Environmental conditions were: 21 ± 2 °C, 55 ± 10% relative humidity and a 12 h light–dark cycle. All animal experiments were conducted in accordance with the UK Home Office Animals (Scientific Procedures) Act 1986 and approved by the Birmingham Ethical Review Subcommittee (PP3501305). Drinking water was supplemented with either corticosterone (100 μg/mL, 0.6% ethanol), or vehicle (0.6% ethanol), with an average consumption of 1.25 mg per day. Mice were sacrificed at the end of 2 weeks’ treatment. Average daily intake of corticosterone was 22.5 µg/g in the mice, roughly equating to administration of 40 mg hydrocortisone or 10 mg of prednisolone per day in an adult [26]. Serum was collected by cardiac puncture and tissues, including whole tibias excised for analysis. Serum calcium and phosphate concentrations were determined by commercial biochemical analysis using an IDEXX automated chemistry analyzer (IDEXX Laboratories, Westbrook, ME, USA).

### Tibia gene expression analysis

Tibias were powdered under liquid nitrogen, and RNA was isolated using innuPREP RNA Mini Kit (Analytikjena, Cambridge) and reverse transcription (Multiscribe, ThermoFisher Scientific) as per the manufacturer’s guidelines. Gene expression was then assessed by commercial TaqMan Gene Expression Assays (ThermoFisher Scientific) using species-specific probe sets by real-time PCR on an ABI7500 system (Applied Biosystems, Warrington, UK). mRNA abundance was normalised to Gapdh. Data were obtained as Ct values and ΔCt determined (Ct target–Ct GAPDH), were expressed as arbitrary units (AU) using the following transformation: (arbitrary units (AU) = 1000 × (2 − Δct)).

### Measurement of FGF23, PTH, P1NP, TRACP 5b and CTX-1

Serum iFGF23 (Quidel, Cat. #60–6600) and cFGF23 (Quidel, Cat. #60–6100) levels were measured using ELISA. Serum PTH was measured using a commercially available sandwich ELISA (Antibodies.com; A78690) in accordance with the manufacturer’s instructions and data expressed as pg/mL. Serum P1NP was determined using a commercially available sandwich ELISA (cat no: AC-33F1, Immunodiagnosticsystems, Tyne & Wear, UK) in accordance with the manufacturer’s instructions and data expressed as ng/mL. Serum TRACP 5b was determined using a commercially available sandwich ELISA (cat no: SB-TR103, Immunodiagnosticsystems, Tyne & Wear, UK) in accordance with the manufacturer’s instructions and data expressed as U/µL.

### *In vitro* studies

MC3T3-E1 subclone 4 mouse preosteoblasts (RRID:CVCL_5440) were used at passages 5–9 and cultured and differentiated as previously described [21]. Osteogenic differentiation was initiated by culturing the cells in a medium containing 50 μg/ml ascorbic acid and 10 mM β-glycerophosphate for 6 days. Upon differentiation, cells were treated with corticosterone (Sigma-Aldrich, Cat. #235135) or hydrocortisone (Sigma-Aldrich, Cat. #H4001) (0–200 nM) for 24 h as noted in results section. *Fgf23* mRNA levels were quantified using SYBR Green-based RT-qPCR (Supplementary Table 3). FGF23 protein levels in the supernatant were measured by ELISA after concentrating the cell supernatant using Pierce Protein Concentrator (PES, 10 K MWCO: Thermo Fisher, Cat. #88527), as described earlier [21]. For GR inhibition studies, cells were pre-treated with 50 nM RU-486 (R&D Systems/Bio-Techne, Catalog #1479) for 1 h, followed by corticosterone treatment. All experiments were performed in triplicate.

### RNA-seq analysis

Differentiated MC3T3 cells were treated with 100 nM corticosterone for 24 h (*n* = 3 biological replicates). RNA was extracted, and sequencing was performed commercially at Novogene using the Illumina platform [21]. Differential gene expression (DEG) analysis was conducted using DESeq2, with adjusted *p*-values (padj < 0.05) and log_2_ fold-change > 1 considered significant. Gene ontology (GO) analysis was performed to identify enriched biological pathways as previously described [21].

### Statistical analysis

Data were presented as mean ± SEM. For the dietary salt intervention study, in which the same participants underwent low-salt, normal-salt, and high-salt dietary conditions, differences across the three conditions were analyzed using repeated-measures one-way ANOVA. For two-group comparisons, unpaired or paired *t*-tests were used as appropriate. Correlation analyses were conducted using Pearson’s correlation coefficient. For in vitro experiments, *n* represents independent biological replicates. For the clinical studies, *n* represents individual participants; for mouse experiments, *n* represents individual mice. All analyses were performed using GraphPad Prism or RStudio.

## Results

**Fig. 1:**
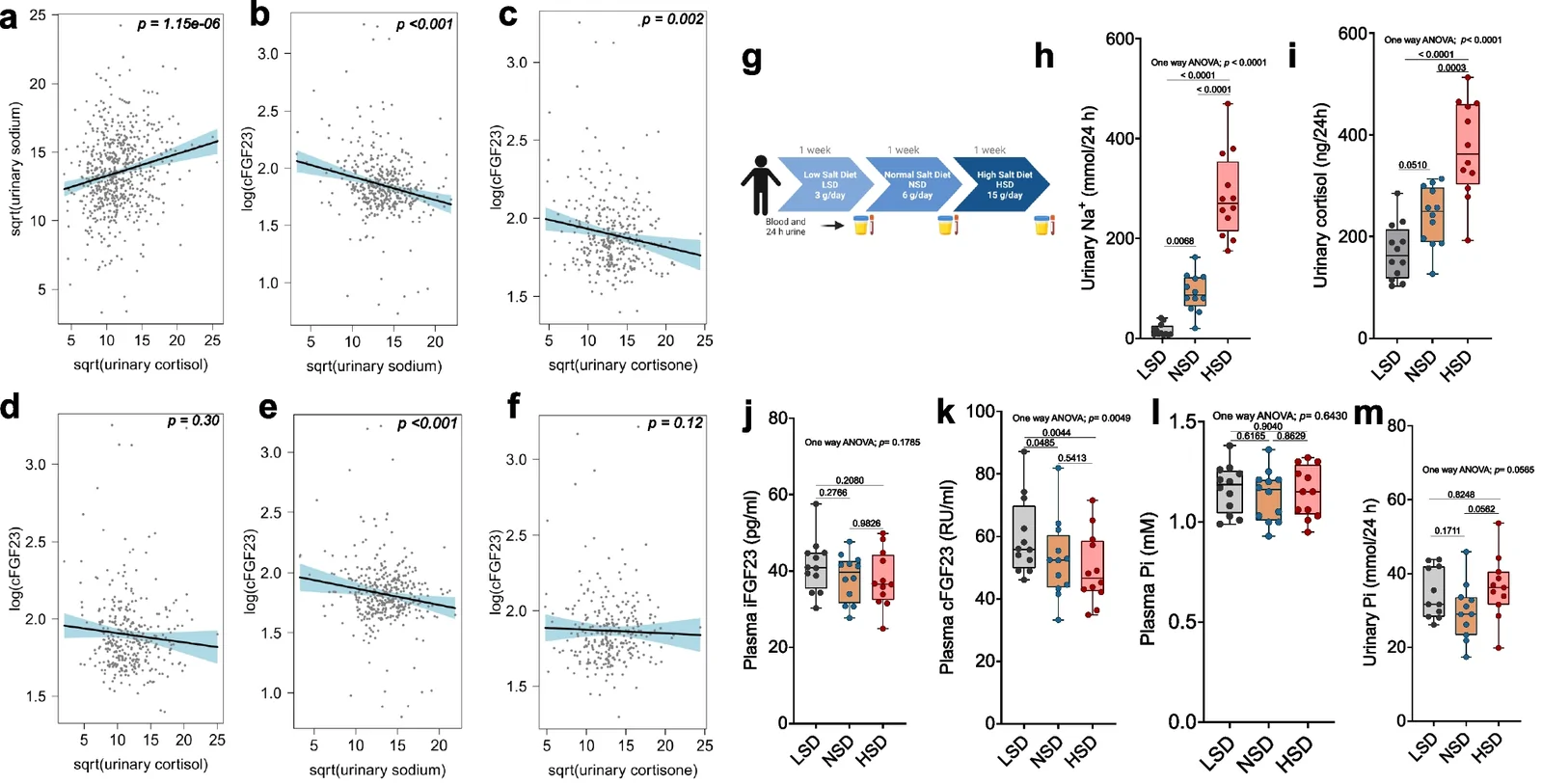
High salt intake in humans elevates cortisol signaling and suppresses FGF23 formation. **a** Positive correlation between 24-h urinary sodium excretion (a marker of dietary salt intake) and urinary cortisol levels in the BKSR cohort. **b** Inverse correlation between 24-h urinary sodium excretion and plasma cFGF23 levels in the BKSR cohort. **c** Inverse correlation between plasma cFGF23 levels and 24-h urinary cortisone levels in the BKSR cohort. **d** Inverse correlation between plasma cFGF23 levels and 24-h urinary cortisol levels in the BKSR cohort. The p-value indicates the statistical significance of the observed correlation. *n* = 321. **e** Multivariable regression analysis showing the association between 24-h urinary sodium excretion and plasma cFGF23 levels after adjustment for age, sex, eGFR, and BMI in the BKSR cohort. **f** Multivariable regression analysis showing the association between 24-h urinary cortisone and plasma cFGF23 levels after adjustment for age, sex, eGFR, and BMI in the BKSR cohort (*p* = 0.12). *n* = 321 for panels A–F. **g** Schematic representation of the dietary salt intervention study design. **h** Urinary sodium excretion after dietary salt intervention with low-salt, normal-salt, and high-salt diets. **i** Urinary cortisol levels. **j** Plasma iFGF23 levels. **k** Plasma cFGF23 levels. **l** Plasma phosphate levels. **m** Urinary phosphate excretion. Data are presented as mean ± SEM. Repeated-measures one-way ANOVA was used to assess differences across the three dietary conditions, followed by Tukey’s post hoc multiple-comparisons test. The *p* value above each panel represents the overall ANOVA, and *p* values above the brackets represent Tukey-adjusted pairwise comparisons

**Fig. 2:**
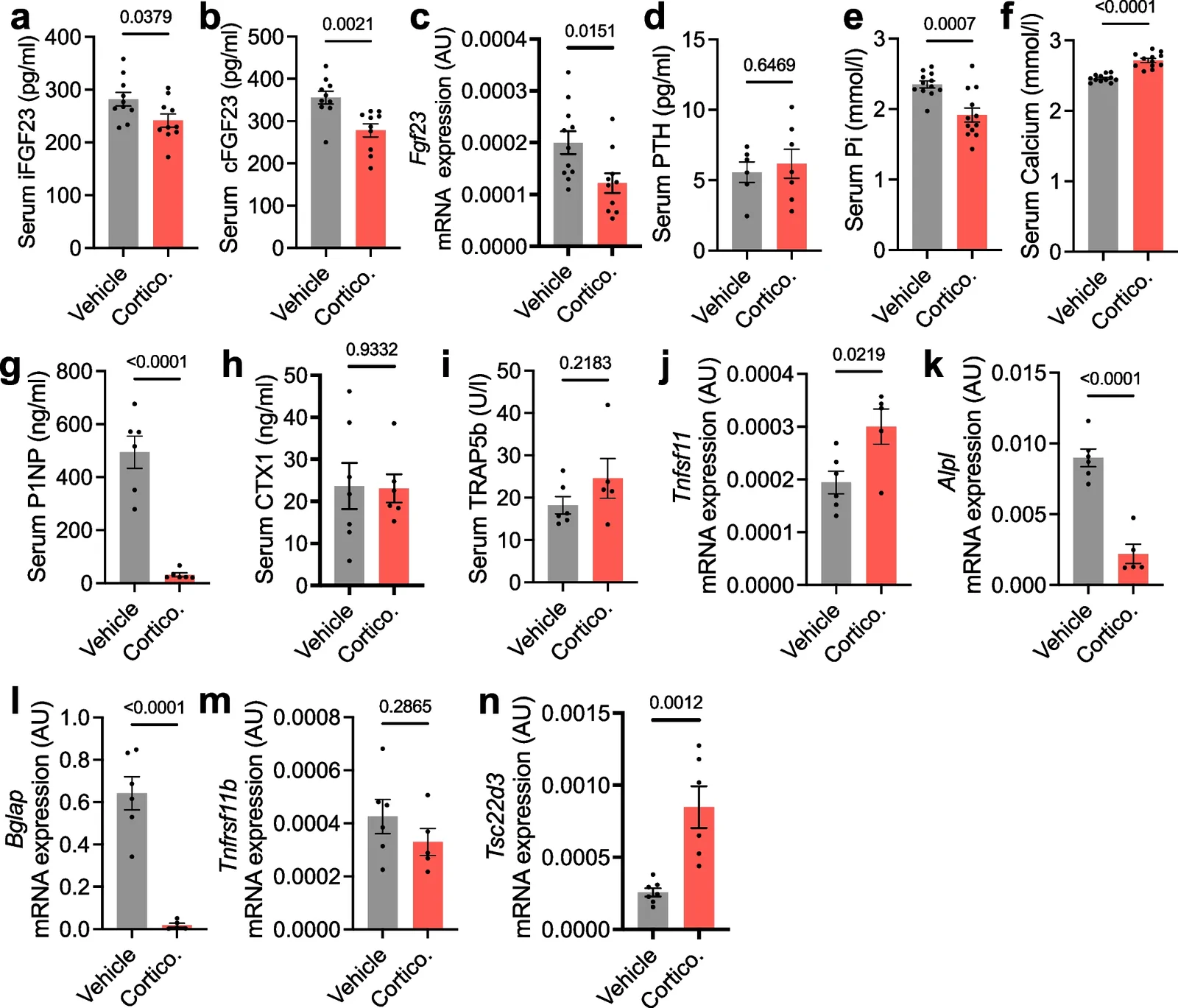
Corticosterone suppresses FGF23 and bone formation in mice. **a** Serum intact FGF23 (iFGF23) and **b** C-terminal FGF23 (cFGF23) levels in C57BL/6 wild-type mice treated orally with corticosterone for 2 weeks (*n* = 10 per group). **c** *Fgf23* mRNA expression in bone and **d** Serum PTH levels following corticosterone treatment (*n* = 6–12 per group). **e** Serum phosphate and **f** serum calcium levels following corticosterone treatment (*n* = 12 per group). **g** Serum procollagen type I N-terminal propeptide (PINP), a marker of bone formation, **h** Serum C-terminal telopeptide of type I collagen (CTX), and **i** Serum tartrate-resistant acid phosphatase 5b (TRAP5b), markers of bone resorption (*n* = 6 per group). **j** *Tnfsf11* (*Rankl*), **k** *Alpl*, **l** *Bglap*, **m** *Tnfrsf11b* (*Opg*), and **n** *Tsc22d3 (Gilz)* mRNA expression in bone (*n* = 5–6 per group). Data are presented as mean ± SEM. Statistical significance is indicated by p-values

**Fig. 3:**
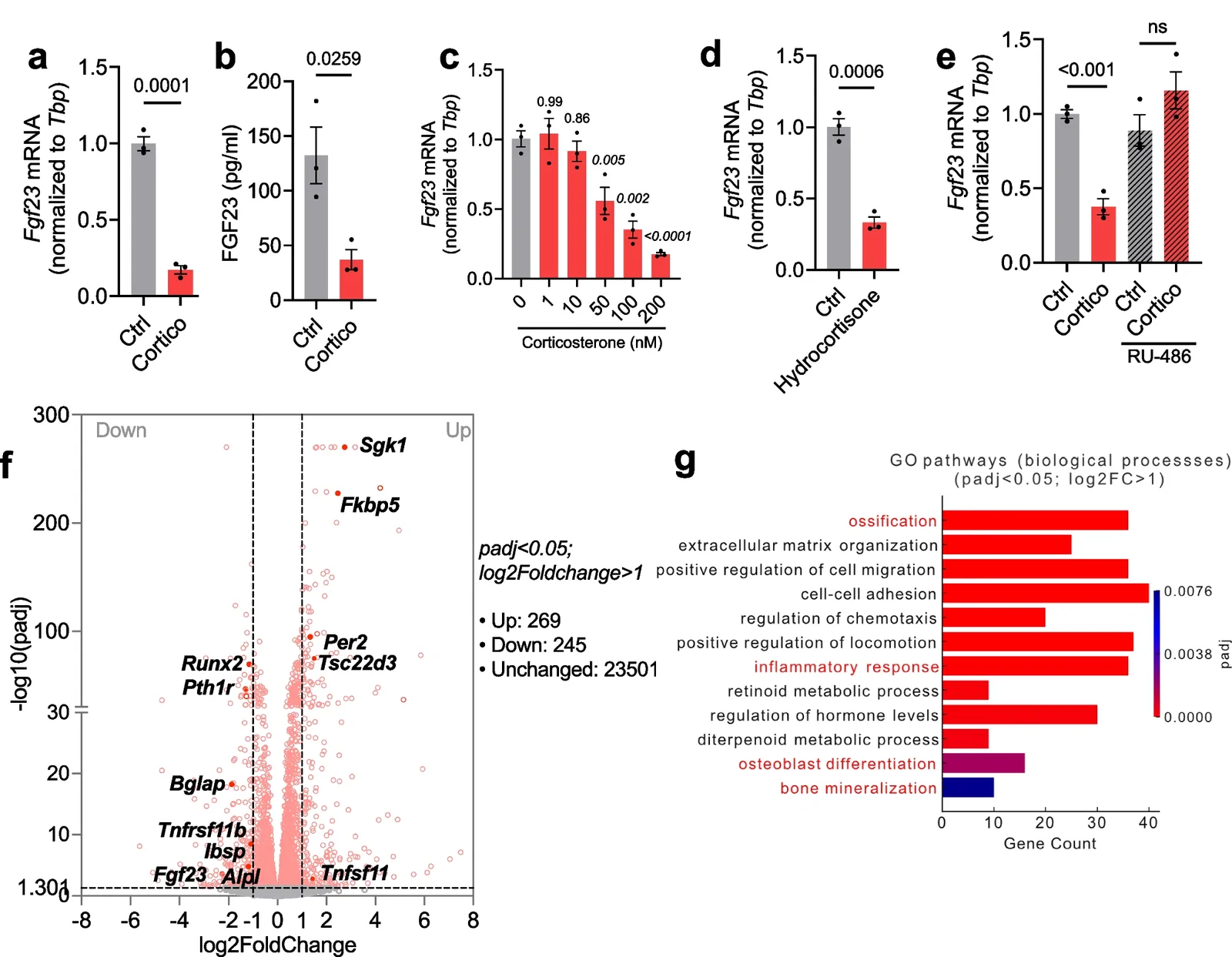
Corticosterone suppresses FGF23 expression through glucocorticoid receptor activation in osteoblasts. **a** *Fgf23* mRNA expression in cell lysates and **b** FGF23 levels in cell supernatants following treatment of MC3T3-E1 osteoblasts with 100 nM corticosterone for 24 h (*n* = 3 per group). **c** Dose-dependent suppression of *Fgf23* mRNA expression following treatment with corticosterone for 24 h **d** *Fgf23* mRNA expression following treatment with 100 nM hydrocortisone for 24 h (*n* = 3). **e** Rescue of corticosterone (100 nM)-induced suppression of *Fgf23* mRNA expression by pretreatment with RU-486, a glucocorticoid receptor (GR) antagonist (*n* = 3). **f** Volcano plot showing differentially expressed genes (DEGs) in MC3T3-E1 osteoblasts treated with 100 nM corticosterone for 24 h. Significantly upregulated and downregulated genes (padj < 0.05; |log2 fold change|> 1) are shown, with selected genes associated with GR signaling, inflammatory responses, and osteoblast-related processes highlighted. **g** Gene ontology (GO) enrichment analysis of biological processes identified among the DEGs. Data are presented as mean ± SEM. Two-group comparisons (a, b, d, e) were analyzed using unpaired two-tailed t-tests. Differences among multiple corticosterone concentrations (c) were analyzed using one-way ANOVA followed by Tukey’s post hoc multiple-comparisons test

1. **High-salt dietary intake is associated with increased cortisol and reduced circulating FGF23.**
  To investigate the association between dietary sodium intake and cortisol signaling, we reanalyzed data from the thoroughly phenotyped population of the Bern Kidney Stone Registry (BKSR), a single-center observational cohort of kidney stone formers [24]. The clinical characteristics of the study population are summarized in Supplemental Table 1. We found that 24-h urinary sodium, a reliable surrogate marker of dietary sodium intake, was associated with 24-h urinary cortisol (Fig. 1a). Further, urinary sodium was negatively associated with plasma FGF23 levels (Fig. 1b). Notably, urinary cortisone and cortisol tended to show an inverse relationship with plasma FGF23; however, only the association with urinary cortisone but not cortisol reached statistical significance (Fig. 1c, d). After adjusting the models for renal function, age, sex and body mass index in multivariable regression, the association with sodium remained whereas the association with cortisone was attenuated and did not remain significant (p = 0.12) (Fig. 1e-f).
  Next, we analyzed serum and urine samples from a prospective crossover-controlled study involving healthy male volunteers [25]. These participants underwent three 7-day dietary interventions: a low-salt diet (LSD, 3 g NaCl/day), a normal-salt diet (NSD, 6 g NaCl/day), and a high-salt diet (HSD, 15 g NaCl/day), with constant potassium intake (Fig. 1g). Dietary sodium intake dose-dependently increased both 24-h urinary sodium excretion (Fig. 1h) and cortisol levels (Fig. 1i). Dietary sodium intake did not statistically significantly suppress plasma iFGF23 levels, although iFGF23 showed a strong trend toward suppression with increasing dietary salt intake (Fig. 1j), whereas plasma cFGF23 levels were dose-dependently suppressed by dietary salt intake (Fig. 1k). Plasma phosphate levels and urinary phosphate excretion did not differ significantly across the dietary interventions (Fig. 1l/m). This dissociation between FGF23 suppression and unchanged phosphate levels suggests that the magnitude of FGF23 reduction may fall below the threshold required to perturb phosphate homeostasis, or that compensatory mechanisms maintain phosphate balance despite reduced FGF23.
2. **Corticosterone suppresses FGF23 levels and alters bone metabolism in mice**
  C57Bl6 wild-type mice were administered corticosterone orally for 2 weeks. At the end of the treatment period, blood samples were collected, and intact FGF23 (iFGF23) and C-terminal FGF23 (cFGF23) levels were measured. We found that corticosterone treatment significantly suppressed both iFGF23 and cFGF23 levels in mice (Fig. 2a, b). Consistent with these changes, corticosterone treatment also significantly reduced *Fgf23* mRNA expression in bone (Fig. 2c), whereas PTH levels were unchanged (Fig. 2d). Corticosterone treatment also significantly reduced serum phosphate levels and increased serum calcium levels (Fig. 2e/f). We next assessed markers of bone formation and resorption. The bone formation marker procollagen type I N-terminal propeptide (PINP) was markedly reduced following corticosterone treatment (Fig. 2g), whereas the bone resorption markers C-terminal telopeptide of type I collagen (CTX) and tartrate-resistant acid phosphatase 5b (TRAP5b) were not significantly altered (Fig. 2h/i).
  We next assessed the expression of genes involved in osteoblast activity and the regulation of bone remodelling. *Tnfsf11 (Rankl)* mRNA expression was significantly increased, whereas *Alpl* and *Bglap* expression were significantly reduced following corticosterone treatment (Fig. 2j–l). *Tnfrsf11b (Opg)* expression was not significantly altered (Fig. 2m). *Tsc22d3/Gilz* (glucocorticoid-induced leucine zipper), involved in GR activation was significantly increased (Fig. 2n). Together, these findings indicate that corticosterone treatment is associated with reduced bone formation, as evidenced by markedly reduced circulating PINP and decreased expression of the osteoblast-associated genes *Bglap* and *Alpl*, whereas the measured markers did not indicate increased bone resorption.
3. **Corticosterone suppresses FGF23 expression through glucocorticoid receptor activation in osteoblasts.** To investigate the mechanisms for FGF23 suppression by corticosterone, in vitro experiments were conducted using MC3T3 cells. Treatment with 100 nM corticosterone for 24 h significantly reduced both cFGF23 levels in the cell supernatant and *Fgf23* mRNA expression in cell lysates (Fig. 3a, b). *Fgf23* mRNA expression was dose-dependently suppressed when cells were treated with 0–200 nM corticosterone (Fig. 3c). Treatment with hydrocortisone (100 nM), a synthetic cortisol analogue, also resulted in a reduction in *Fgf23* mRNA expression (Fig. 3d). Pretreatment with RU-486 (GR blocker) completely rescued the corticosterone-induced suppression of *Fgf23* mRNA, suggesting that the effects of corticosterone on *Fgf23* expression are mediated through GR activation (Fig. 3e).

RNA-seq analysis was performed on MC3T3 cells treated with 100 nM corticosterone for 24 h. Differential gene expression analysis revealed 269 upregulated and 245 downregulated genes (padj < 0.05; log_2_FoldChange > 1), visualized in a volcano plot (Fig. 3f). Gene ontology (GO) analysis of biological processes revealed key pathways, including "ossification," "inflammatory response," "osteoblast differentiation," and "bone mineralization." (Fig. 3g). Further information is provided in the supplementary datasets 1 and 2 for DEGs and in the complete list of GO pathways, respectively. Genes associated with direct corticosteroid signaling, including Tsc22d3/Gilz (glucocorticoid-induced leucine zipper), *Sgk1*, *Fkbp5*, and *Per1*, were significantly upregulated, highlighting GR activation [27] (Fig. 3f/g). Anti-inflammatory pathways were prominently modulated, with a strong upregulation of classical corticosteroid-responsive genes such as *Hp* (Haptoglobin), *Orm1/2*, and *Serpine1* and a downregulation of *Ednra* [28] (Fig. 3g; supplementary dataset 2). Simultaneously, genes involved in osteoblast differentiation, such as *Runx2*, *Pth1r*, and *Ibsp*, were downregulated, indicating reduced osteoblast activity and extracellular matrix remodeling [29]. Downregulation of *Bglap, Ibsp, Alpl, Tnfsf11/Rankl, Tnfrsf11b/Opg* further supported reduced osteoblast activity. These transcriptomic findings were consistent with reduced expression of osteoblast-associated genes in the tibia of corticosterone-treated mice (Supplemental Table 2). These findings provide evidence that corticosteroids suppress FGF23 through combined GR signaling, anti-inflammatory effects, and suppression of osteoblast activity.

## Discussion

Our study provides evidence for a potential endocrine cascade by which high salt intake elevates cortisol signaling and suppresses FGF23 levels, revealing non-classical pathophysiological consequences of high-salt diets on systemic health.

We measured 24-h urinary cortisol for its accuracy in reflecting systemic cortisol secretion, overcoming the limitations of diurnal variation and inter-individual variability in serum levels [30]. Prior interventional studies with smaller sample sizes suggested that dietary salt intake modulates urinary cortisol levels [5, 6]. While real-world dietary sodium variations are less extreme than those in interventional settings, our retrospective analysis showed that even among individuals on random diets, urinary cortisol positively correlates with dietary salt intake. Two possible mechanisms may explain the cortisol rise with high salt intake. First, both aldosterone and cortisol are synthesized in the adrenal cortex, share initial biosynthetic steps, and their levels are affected due to competition for shared precursors and enzymes in the context of salt intake. Suppression of RAAS activity by high salt reduces the demand for aldosterone synthesis, which depends on the enzyme *CYP11B2*. This reduction may divert precursors such as 11-deoxycorticosterone toward cortisol synthesis, potentially increasing cortisol levels [1, 31]. Second, a recent study in mice suggest high-salt intake elevates corticosterone levels due to activated hypothalamus-pituitary (HPA) axis and stress response [32].

We found that high salt-mediated cortisol signaling was negatively associated with plasma FGF23. In the retrospective analysis of individuals consuming random diets, urinary cortisone correlated more strongly with FGF23 than cortisol, likely due to its higher stability. While the association did not remain statistically significant after adjustment, potential residual confounding by renal function cannot be excluded. Notably, in a controlled dietary salt intervention in young adults, high salt intake consistently increased urinary cortisol and suppressed plasma FGF23, supporting this relationship under experimental conditions. The more pronounced suppression of cFGF23 compared to iFGF23 under high-salt conditions may suggest that cortisol modulates not only FGF23 production but also its proteolytic cleavage, though this warrants dedicated investigation.

FGF23 is primarily recognized for its role in phosphate and vitamin D homeostasis, but its connection to salt metabolism is of emerging interest [14–16, 19, 20]. Aldosterone, a key hormone of the RAAS, directly enhances FGF23 production in bone via mineralocorticoid receptors [14, 19]. This was linked to a potential mechanism for FGF23 regulation through dietary salt intervention in previous studies [20]. Our findings suggest that elevated cortisol and reduced aldosterone may collectively contribute to FGF23 suppression in response to high salt intake. However, our study provides limited direct evidence for high salt-induced cortisol suppressing FGF23 levels in humans and highlights the need for further investigation. Furthermore, dedicated in vivo experiments are warranted to determine the relative contributions of cortisol and aldosterone to FGF23 regulation under a high-salt diet. 

Previous studies with potent glucocorticoids dexamethasone and prednisolone have shown both stimulatory and inhibitory effects on FGF23 levels in mice [22, 23]. In contrast, our results indicate that the naturally occurring hormone corticosterone suppresses FGF23 production in mice and osteoblast cells via GR activation, anti-inflammatory signaling pathways, and transcriptional repression of osteoblast activity. Our findings therefore support a context-dependent role of glucocorticoid signaling in the regulation of FGF23 and extend previous observations by demonstrating suppression of FGF23 by the endogenous glucocorticoid corticosterone in both osteoblasts and mice. 

Importantly, our additional in vivo analyses support an effect of corticosterone on bone FGF23 expression and bone formation. Corticosterone-treated mice showed reduced bone *Fgf23* mRNA expression together with reduced serum PINP and decreased expression of the osteoblast-associated genes *Bglap* and *Alp*. These changes were accompanied by reduced serum phosphate and increased serum calcium, whereas PTH and the measured markers of bone resorption were unchanged. Together with MC3T3 osteoblast experiments, these findings support the effect of glucocorticoid exposure on reduced osteoblast activity and suppression of FGF23 expression. 

The possibility of a direct effect of glucocorticoids on renal phosphate reabsorption is also important. Glucocorticoids have been shown to reduce proximal tubular sodium-dependent phosphate transport, including NaPi-2a, thereby promoting phosphaturia and contributing to hypophosphatemia independently of FGF23 and PTH [33–35]. Our additional mouse experiments showed that corticosterone reduced serum phosphate despite modestly reduced FGF23 levels. These findings raise the possibility that a direct renal effect of glucocorticoids on phosphate reabsorption may contribute to the observed hypophosphatemia, potentially counteracting the phosphate-retaining effect expected from reduced FGF23.

The interplay between dietary salt, cortisol signaling, and FGF23 may have important systemic implications. Both high salt intake and elevated cortisol levels contribute to hypertension, autoimmune diseases, inflammation and metabolic syndrome, bone loss and fracture risks, therefore they may create a vicious cycle and amplify the burden of chronic diseases [1, 3]. Future studies are warranted to explore pharmacological strategies to counteract glucocorticoid-mediated effects of high-salt diets on bone and cardiovascular health. Furthermore, both cortisol and FGF23 are used in many diagnostic tests [30, 36]. Our results suggest that variations in dietary salt intake may influence the interpretations of these measurements.

We acknowledge limitations of our study. First, the dietary intervention study included only 12 participants and was originally designed to investigate renal electrolyte handling rather than FGF23 biology. Therefore, the present analyses should be considered exploratory. In addition, plasma PTH, active vitamin D, and calcium were not measured during the original human dietary intervention, and no samples remain available for retrospective measurement. Therefore, the contribution of these mineral-regulatory factors to the FGF23 response to dietary salt cannot be determined in the present study. Second, although the controlled dietary intervention and experimental studies provide complementary evidence for a role of glucocorticoid signaling in FGF23 regulation, the observational association between urinary cortisone and FGF23 was attenuated after adjustment for eGFR. Therefore, the extent to which glucocorticoid signaling contributes independently to dietary salt-induced regulation of FGF23 in humans remains to be established.

In conclusion, our study provides evidence for a potential endocrine link between dietary salt intake, cortisol signaling, and FGF23 regulation. These findings suggest an unconventional role of dietary salt in systemic health and highlight the need for further study.

## Supplementary Information

Below is the link to the electronic supplementary material.

Supplementary file1 (PDF 158 KB)

Supplementary file2 (XLSX 8024 KB)

Supplementary file3 (XLSX 543 KB)

## Data Availability

The raw RNA-seq data used in this publication have been deposited in NCBI's Gene Expression Omnibus and are accessible through accession number GSE304562. All data are available from the authors upon request.
