# Supplementary material for: High-salt diet modulates endocrine regulation between cortisol and FGF23
Source: Pflugers Arch. 2026 Sep 19;478(10):81. doi: 10.1007/s00424-026-03211-x (PMC13588846; doi:10.1007/s00424-026-03211-x)
Supplement: Supplementary file 1 [file 424_2026_3211_MOESM1_ESM.pdf]

High-salt diet modulates endocrine regulation between cortisol and FGF23

Matthias B. Moor[1,2], Michael S. Sagmeister[3], Ana Crastin[4,5], Klaudia Kopper[2,6], Antoinette Pechère-Bertschi[7,8], Rowan S. Hardy[4,5], Eric Feraille[2,7,8], Daniel G. Fuster[1,2], Johannes Loffing[2,6], Ganesh Pathare[2,6,9,10]

[1]Department of Nephrology and Hypertension, Inselspital, Bern University Hospital, University of Bern, Bern, Switzerland.
[2]Swiss National Centre of Competence in Research "Kidney Control of Homeostasis", Zurich, Switzerland
[3]Institute of Applied Health Research, College of Medicine and Health, University of Birmingham, Birmingham B15 2TT, UK.
[4]MRC Arthritis Research UK Centre for Musculoskeletal Ageing Research, University of Birmingham, Birmingham, B15 2TT, UK.
[5]Department of Biomedical Sciences, School of Infection, Inflammation & Immunology, College of Medicine and Health, University of Birmingham, Birmingham B15 2TT, UK.
[6]Institute of Anatomy, University of Zurich, Zurich, Switzerland
[7]Service of Nephrology and Hypertension, University Hospital Geneva, Geneva, Switzerland.
[8]Department of Cell Physiology and Metabolism, University of Geneva, Geneva, Switzerland.
[9]Institute of Veterinary Anatomy, University of Zurich, Zurich, Switzerland
[10]Bone-Kidney Axis and Regeneration Laboratory, Dept. of Infectious Diseases and Public Health, City University of Hong Kong, Kowloon Tong, Hong Kong SAR China

***Corresponding author**:
Prof. Ganesh Pathare, BVSc, PhD
Email: gpathare@cityu.edu.hk

Keywords: High-salt-diet, cortisol (corticosterone), FGF23, bone and kidney

Competing Interests: The authors declare no potential conflicts of interest.

This supplementary pdf contains:
Supplementary tables

Characteristic	N	men<br>N = 233[1]	women<br>N = 88[1]	p-value[2]
BMI	321	26.9 (24.5, 29.6)	25.7 (22.9, 30.9)	0.213
Measured GFR	321	115 (97, 130)	101 (85, 125)	0.011
Age	321	48 (37, 57)	45 (36, 56)	0.351
Systolic BP	232	137 (126, 148)	129 (117, 142)	0.005
Unknown		68	21	
Diastolic BP	232	90 (82, 97)	85 (78, 93)	0.066
Unknown		68	21	
Cortisone 24h	321	175 (138, 233)	137 (89, 192)	<0.001
Cortisol 24h	319	135 (96, 191)	111 (74, 157)	0.002
Unknown		1	1	
c-FGF23	321	69 (53, 89)	91 (62, 129)	<0.001

[1] Median (Q1, Q3)

[2] Wilcoxon rank sum test

Supplemental Table 1. Patient characteristics of the Bern Kidney Stone Registry (BKSR). Patient characteristics are presented separately for men and women. Continuous variables are expressed as median (Q1, Q3). p-values were calculated using the Wilcoxon rank-sum test for comparisons between men and women. The number of participants with available measurements is indicated for each characteristic; the number of participants with missing data is indicated as “Unknown.” N = 321 for the overall cohort, including 233 men and 88 women. For 24-hour urinary cortisol, measurements were available in 319 participants.

Gene	Function	Mouse tibia	MC3T3-E1 cells
Tsc22d3 **(*Gilz*)** (glucocorticoid-induced leucine zipper)	Glucocorticoid-responsive gene involved in GR signaling and regulation of inflammatory and cellular responses	↑	↑
Bglap **(*Ocn*)** Bone γ-carboxyglutamate protein (osteocalcin)	Glucocorticoid-responsive osteoblast-associated gene involved in bone formation and mineralization	↓	↓
Alpl Alkaline phosphatase	Osteoblast-associated enzyme involved in bone formation and extracellular matrix mineralization	↓	↓
Tnfsf11 **(*Rankl*)** TNF receptor superfamily member 11	Osteoblast-derived factor that promotes osteoclast differentiation and bone resorption	↑	↑
Tnfrsf11b **(*Opg*)** TNF receptor superfamily member 11b	Osteoblast-derived decoy receptor for RANKL that inhibits osteoclast differentiation and bone resorption	↓(ns)	↓

Supplemental Table 2. Common genes in mouse tibia and MC3T3-E1 osteoblasts following corticosterone treatment. Gene expression in mouse tibia was assessed by qRT-PCR, whereas gene expression in MC3T3-E1 osteoblasts was assessed by RNA-seq following corticosterone treatment. ↑: significantly increased expression; ↓: significantly decreased expression; ns: statistically not significant; *Bglap* is included among both glucocorticoid-responsive and bone formation-associated genes.

Gene	Primer	Sequence (5′–3′)
Mouse *Fgf23*	Forward	TTTCCCAGGTTCGTCTAGG
Mouse *Fgf23*	Reverse	CTCGCAGGTGACTCTCAG
Mouse *Tbp*	Forward	ACCCTTCACCAATGACTCCTATG
Mouse *Tbp*	Reverse	TGACTGCAGCAAATCGCTTGG

Supplemental Table 3. SYBR Green primer sequences used for qRT-PCR in MC3T3-E1 osteoblasts.
